# Sensitivity Enhancement of Resistive Ethanol Gas Sensor by Optimized Sputtered-Assisted CuO Decoration of ZnO Nanorods

**DOI:** 10.3390/s23010365

**Published:** 2022-12-29

**Authors:** Hadi Riyahi Madvar, Zoheir Kordrostami, Ali Mirzaei

**Affiliations:** 1 Department of Electrical and Electronic Engineering, Shiraz University of Technology, Shiraz 71557-13876, Iran; 2 Research Center for Design and Fabrication of Advanced Electronic Devices, Shiraz University of Technology, Shiraz 71557-13876, Iran; 3 Department of Materials Science and Engineering, Shiraz University of Technology, Shiraz 71557-13876, Iran

**Keywords:** ZnO, CuO, ethanol, gas sensor, sensing mechanism

## Abstract

In this study, sputtered-assisted CuO-decorated ZnO nanorod (NR) gas sensors were fabricated for ethanol gas sensing studies. CuO nanoparticles have been successfully formed on ZnO nanorods by means of a physical process as the decorative metallic element. The amount of decoration affecting the sensor’s performance has been optimized. Cu layers with different thicknesses of 5, 10, and 20 nm were deposited on hydrothermally grown ZnO NRs using the sputtering technique. Upon subsequent annealing, Cu was oxidized to CuO. The gas sensing studies revealed that the sensor with an initial Cu layer of 5 nm had the highest response to ethanol at 350 °C. The sensor also showed good selectivity, repeatability, and long-term stability. The enhanced ethanol sensing response of the optimized gas sensor is related to the formation of p-n heterojunction between p-type CuO and n-type ZnO and the presence of the optimal amount of CuO on the surface of ZnO NRs. The results presented in this study highlight the need for optimizing the amount of Cu deposition on the surface of ZnO NRs in order to achieve the highest response to ethanol gas.

## 1. Introduction

Today, human activities driven by the increase in global industrial processes produce toxic chemicals, including toxic gases and volatile organic compounds (VOCs), which are among the main worldwide problematic issues [1]. According to the World Health Organization (WHO), every year about 4.2 million deaths occur as a result of air pollution, with an increasing percentage of these deaths linked to VOCs. Thus, the detection of VOCs is very important and challenging [2]. Ethanol is known as one of the most important VOCs and is widely used in different industries, for instance, fuel processing, beverages, etc. Thus, the reliable detection of ethanol is of great interest to researchers [3].

Metal oxides [4], as well as metal nitrides [5], can be used for the detection of gases. For example, a memristor-based NO gas sensor using metal oxides was reported to have excellent gas sensing performance with extremely fast response times (<1 s) and recovery times (90 ns) at room temperature [6]. In another study, a SnO_2_-based memristor showed high sensing performance towards ethanol gas in terms of high response and fast dynamics [7]. However, currently, the most widely used gas sensors are resistive-based. In particular, resistive-type gas sensors, based on metal-oxide semiconductors (MOSs), are one of the most commonly used sensing devices for monitoring different VOCs. MOS-based sensors have many advantages, such as fast response time, high sensitivity to target gases, small dimensions, portability, cost-effective fabrication, ease of use, and real-time detection [8]. Different MOSs are used for the realization of resistive gas sensors. ZnO is one of the top choices for detecting toxic and harmful gases because of its superior physicochemical properties. ZnO is an n-type II-VI semiconductor with a wide direct bandgap (3.37 eV), large excitation binding energy (60 meV), and high electron mobility (~400 cm^2^ V^−1^s^−1^), which are interesting for gas sensing applications. In addition, ZnO has the advantages of biocompatibility, chemical stability, environmental friendliness, low synthesis cost, etc [9]. There are some strategies to enhance the gas sensing properties of ZnO gas sensors in terms of selectivity and sensing temperature. A common way to modify the sensing performance of ZnO is to add other MOSs or metals in the form of composites, decorations, or doping [10], through various chemical or physical methods.

In this context, Hu et al. [11] reported an acetone gas sensor using a ZnO/ZnFe_2_O_4_ core-shell hollow microsphere that was obtained through a one-pot hydrothermal method. Anajafi et al. [12] reported the acetone sensing behavior of a p-SmFeO_3_/n-ZnO nanocomposite prepared by a chemical route. ZnO nanofibers and perovskites were used for H_2_S gas sensing [13]. Saito et al. [14] reported a highly sensitive isoprene gas sensor using ZnO nanostructures loaded with Au nanoparticles (NPs). Bhowmick et al. [15] reported a selective and sensitive CO_2_ gas sensor based on CuO/ZnO thin film. Zhang et al. [16] reported a butanol gas sensor based on Bi_2_MoO_6_ porous microspheres and ZnO nanosheet composites with good response at 270 °C. Liu et al. introduced a fast-responding toluene gas sensor based on a hollow NiO/ZnO p-n heterostructure with a working temperature of 300 °C [17]. In addition, morphology engineering to obtain high surface area nanostructures such as nanowires, nanofibers, nanorods (NRs), and other morphologies is a popular technique to enhance gas sensing properties. For example, Jia et al. [18] used ZnSnO_3_ hollow microspheres synthesized by the one-pot template-free method for ethanol sensing properties. Additionally, combining morphology engineering and the creation of composite materials is sometimes a good way to improve gas sensing properties. For example, Guo et al. [19] enhanced the ethanol sensing performance of ZnSnO_3_ hollow microspheres by adding CNTs in a hydrothermal process. In other studies, decorated ZnO NRs with PbS quantum dots have been used for room-temperature ethanol gas sensing [20].

Recently, physical methods such as sputtering have become popular for the realization or decoration of gas sensing materials. This is due to the simplicity of this technique and the existence of exact control over the deposited film. In this context, Drmosh et al. [21] synthesized Au-decorated ZnO/Ag core-shell films for acetone gas sensing applications. The thickness of the Au layer was adjusted between 3 and 34 nm by DC sputtering. Liang et al. synthesized the Fe_2_O_3_-ZnO composite NRs by combining the hydrothermal growth of Fe_2_O_3_ NRs with sputtering deposition of a thin ZnO layer for acetone gas sensing [22]. Kwon et al. [23] used the sputtering technique for the decoration of Au NPs on ZnO/TiO_2_ NRs for NO_2_ gas sensing studies. In another study, Jaiswal et al. [24] presented a NO_2_ gas sensor based on CdTe-functionalized ZnO-filled porous Si, where both CdTe and ZnO were fabricated using RF-magnetron sputtering. De Lima et al. [25] synthesized rGO-ZnO composites using a combination of RF-magnetron sputtering and the hydrothermal method for ozone sensing at very low concentrations.

P-type CuO with a bandgap of (1.2–1.8 eV) and good physical and electrical properties is a suitable candidate to combine with ZnO to improve its sensing performance [26]. This is mainly due to the catalytic effect of CuO as well as the formation of the p-n heterojunctions between CuO and ZnO. In the literature, some research combines ZnO with CuO to obtain enhanced sensing applications. Zhang et al. [27] reported an ethanol gas sensor based on flower-like p-CuO/n-ZnO heterojunction NRs. Cheng et al. [28] prepared a ZnO/CuO heterojunction for ammonia gas sensing application at room temperature. Zhao et al. [29] synthesized CuO-decorated ZnO nanowires for ethanol gas sensing applications. ZnO/CuO nanocomposites have also been used for highly sensitive and selective H_2_S gas sensing applications [30]. Lee et al. [31] introduced an acetone gas sensor based on ZnO-CuO core-shell structures. Moreover, Wang et al. [32] fabricated a sensitive H_2_S gas sensor based on a ZnO/CuO composite. However, in most studies, there is no systematic investigation and optimization of CuO decoration or ZnO in the sensing material.

Inspired by the above facts, in this paper, a selective and sensitive ethanol gas sensor based on the decoration of CuO NPs on ZnO NRs is presented. First, we used the hydrothermal method to synthesize uniform ZnO NRs, and then a thin Cu layer was deposited on the ZnO NRs by sputtering as a physical deposition method followed by annealing for the formation of isolated CuO NPs on the surface of ZnO NRs. The ethanol gas sensing studies showed that the presence of CuO NPs can significantly improve the performance of a ZnO NR gas sensor in terms of response, selectivity, and sensing temperature.

## 2. Materials and Methods

### 2.1. Starting Materials

Zinc nitrate hexahydrate (Zn${\left({\mathrm{NO}}_{3}\right)}_{2}.6H_{2}O.98\%$) was obtained from Samchun Company, and sodium hydroxide (NaOH, 98%) was obtained from Merk Company. Deionized water was used in all synthesis and fabrication processes.

### 2.2. Synthesis of ZnO NRs

**Figure 1:**
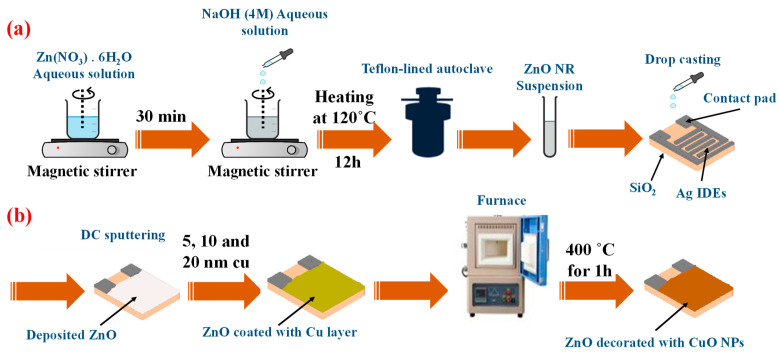
Schematic of preparation of (**a**) pristine ZnO NRs and (**b**) CuO-decorated ZnO NRs.

Figure 1 schematically shows the synthesis process of ZnO NRs. First, 80 mL of 0.044 M solution of zinc nitrate hexahydrate was dissolved in DI water and stirred for 30 min at room temperature. Then, 10 mL of 4 M solution of NaOH was added to the above solution, and the resulting solution was stirred for 1 h. In the next step, the solution was ultrasonicated for 15 min, and then the solution was transferred into a Teflon-lined autoclave and heated for 12 h at 120 °C. The final, white-colored product was washed several times with DI water. The purification process includes ultrasonication (15 min) and centrifugation (10 min at 4000 rpm), which were repeated several times to completely remove the impurities. Figure 1a shows the schematic of the preparation of pristine ZnO NRs and deposition on the sensor substrate. Formation mechanism of ZnO NRs can be shown by Equations (1)–(5) [33,34]:

Zn(NO_3_)_2_.6H_2_O + H_2_O → Zn^2+^_(s)_ + NO_3_^−^_(s)_ (1)

NaOH + H_2_O → Na^+^_(s)_ + OH^−^_(s)_ (2)

Zn^2+^_(s)_ + NO_3_^−^_(s)_ + Na^+^_(s)_ + OH^−^_(s)_ → Zn (OH)_2(s)_ + 2NaNO_3(s)_ (3)

Zn (OH)_2(s)_ → ZnO_(s)_ + H_2_O_(L)_ (4)

(5) $$\mathrm{ZnO}\;{\mathrm{nanoparicles}}_{\left(s\right)}\;\overset{High\;T\;and\;high\;P}{\to }\mathrm{ZnO}\;{\mathrm{nanorods}}_{\left(s\right)}$$

### 2.3. Sensor Fabrication

**Figure 2:**
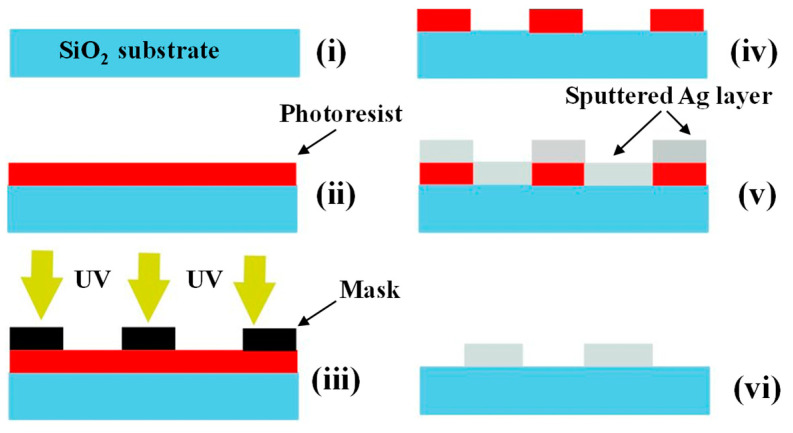
Ag IDEs photolithography process. (**i**) substrate (**ii**) photoresist on substrate (**iii**) UV illumination (**iv**) generated pattern (**v**) sputtered Ag layer (**vi**) final electrodes on substrate.

Alumina (Al_2_O_3_) substrate has been used as the substrate (dimensions of 17 × 21 mm^2^) because of its stability at high temperatures. Silver electrodes were deposited on the substrate to form the interdigital electrodes (IDEs). To pattern the electrodes, first a thin layer of positive photoresist (S1813) was deposited on the substrate using a lab spin coater. Then, the deposited photoresist layer was patterned by UV exposure through a printed mask, and the sample was dipped into the developing solution. In the next stage, a 200 nm thick silver layer was deposited on the surface of the alumina substrate by using DC sputtering method. Finally, the remaining photoresist was removed by acetone washing. The fabrication process for Ag IDEs is shown schematically in Figure 2. The sensing area is 1.4 × 1 cm^2^ containing 10 fingers with 600 µm gap spacing and 500 µm finger widths.

### 2.4. CuO Decoration

**Figure 3:**
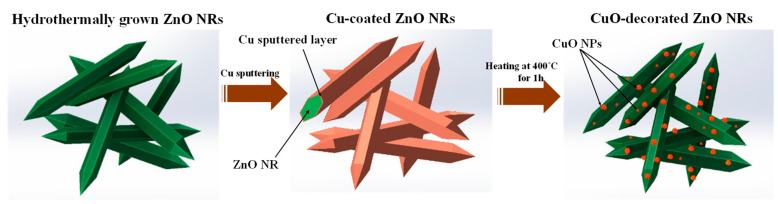
The CuO nanoparticles formation on the surface of ZnO NRs using sputtering followed by annealing.

Figure 1b shows the schematic of the preparation process of CuO-decorated ZnO NRs. In order to produce a highly sensitive and selective ethanol gas sensor, the synthesized ZnO nanorods were decorated with CuO nanoparticles. First, 100 μL of the ZnO NRs suspension was dropped onto a substrate equipped with Ag electrodes. Using sputtering deposition, different thicknesses of copper (5, 10, and 20 nm) were deposited on ZnO NRs. The samples without the Cu (pure ZnO NRs) and with Cu layer thicknesses of 5, 10, and 20 nm were coded as ZC0, ZC5, ZC10, and ZC20, respectively. The sputtering rate was adjusted to approximately 1.3 Å/s and the pressure was 5.4 × $10^{-3}$ bar. After the deposition, to oxidize the copper layer, the samples were placed in a laboratory furnace and annealed at 400 °C for 1 h. Similar annealing process has been used in [35] to obtain the copper oxide. As schematically shown in Figure 3, the annealing stage causes further oxidation of the copper layer and the formation of CuO nanoparticles.

### 2.5. Gas Sensing Measurements

The sensing performance of the fabricated sensor samples was measured using a lab gas sensing set-up. A Victor 86D multimeter connected to a PC was used for continuous recording of the resistance. The working temperature of the sensor was adjusted by a tunable heater. The amount of liquid VOC injected into the chamber to create a specific concentration of gas was calculated according to this equation [36,37]:

(6) $$V_{\mathrm{eth}}=\frac{V\times C\times W\times T_{\mathrm{stan}}\times 10^{-3}}{22.4\times \rho \times T_{\mathrm{sh}}}$$

where V_eth_ (μL) is the volume of ethanol (C_2_H_5_OH), V (L) is the volume of the test chamber, C is the purposed concentration of gas, W is the molar weight of VOC, Tstan (K) is the temperature of standard condition, T_sh_ (K) is the ambient temperature, ρ (g/cm^3^) is the density of VOC, and 22.4 is the molar volume of the standard gas.

The gas sensor response is defined as R_a_/R_g_ for reducing gases and R_g_/R_a_ for oxidizing gases, where R_a_ is the sensor resistance in air and R_g_ is the resistance in the presence of the target gas. In this paper, since ethanol is a reducing gas, the R_a_/R_g_ formula for studying responses of fabricated gas sensors has been used. The response time corresponds to the time required for the sensor resistance to reach 90% of the final resistance after the injection of the ethanol gas (R_a_ to R_a_−0.9(R_a_−R_g_)), and the recovery time is the time required for the sensor resistance to reach 90% of the initial resistance after the removal of the ethanol gas (R_g_ to R_g_ + 0.9(R_a_−R_g_)).

## 3. Results and Discussion

### 3.1. Morphological, Chemical, and Structural Characterizations

The morphology of the pristine ZnO sample was studied by scanning electron microscopy (SEM, TESCAN-Vega3), and the ZnO/CuO samples were studied by field emission scanning electron microscopy (FE-SEM, MIRA3 TESCAN-XMU) equipped with energy dispersive X-ray spectroscopy (EDX) used for chemical analysis of the samples. X-ray diffraction (XRD; Cu Kα radiation λ = 1.54056 Å) studies were performed using a Bruker D8-ADVANCED X-ray diffractometer to study the phase and crystallinity of the samples.

**Figure 4:**
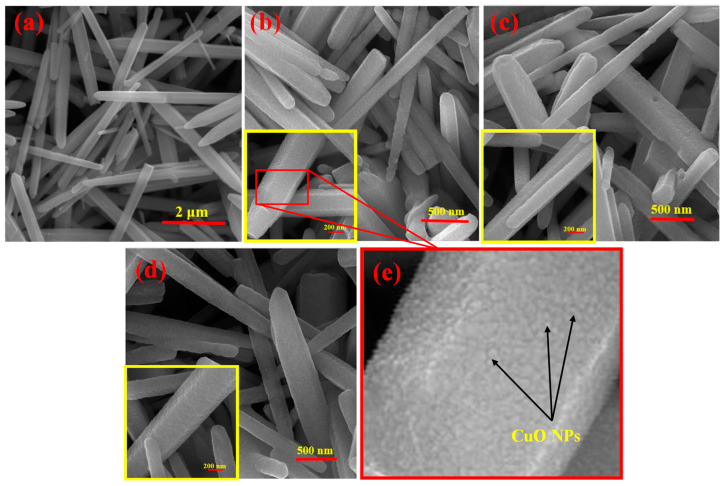
SEM image of (**a**) pristine ZnO NRs and FE-SEM images of CuO-decorated ZnO NRs with an initial Cu layer thickness of (**b**) 5 nm (ZC5), (**c**) 10 nm (ZC10), (**d**) 20 nm (ZC20). Insets show higher magnification of FE-SEM images. (**e**) The magnified part in the inset of (**b**).

Figure 4a shows an SEM image of the pristine ZnO NRs, which shows smooth morphology and a high aspect ratio, demonstrating the successful synthesis of ZnO NRs. In addition, Figure 4b–d show the FE-SEM images of CuO-decorated ZnO NRs with initial Cu thicknesses of 5, 10, and 20 nm, respectively. The corresponding higher-magnification FE-SEM images are also shown in the inset of Figure 4a–d. The magnified part in the inset of Figure 4b clearly reveals the increased roughness of the NRs surfaces because of the presence of the CuO nanostructures as illustrated in Figure 4e.

**Figure 5:**
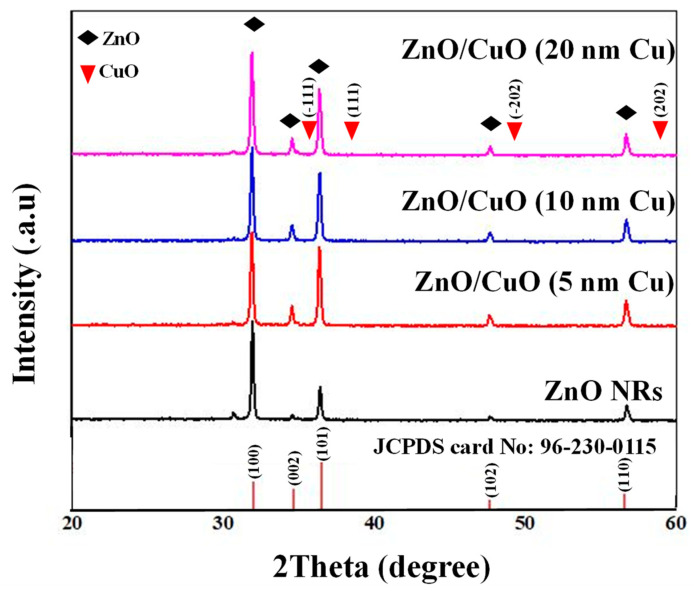
XRD patterns of pristine and CuO-decorated ZnO NRs.

The XRD patterns of pristine and CuO-decorated ZnO NRs are depicted in Figure 5. The XRD pattern of pristine ZnO NRs shows peaks of (100), (002), (101), (102), and (110) crystalline planes of hexagonal ZnO (JCPDS Card No. 96-230-0115). Additionally, there is no other peak related to impurities or undesired phases, showing the success of the employed synthesis procedure and the high purity of the starting materials. For CuO-decorated ZnO NRs, similar peaks were observed. The lack of CuO in XRD patterns can be due to the very low amount of CuO below the detection limit of XRD. Thus, to further study the chemical composition, we used EDX analysis.

**Figure 6:**
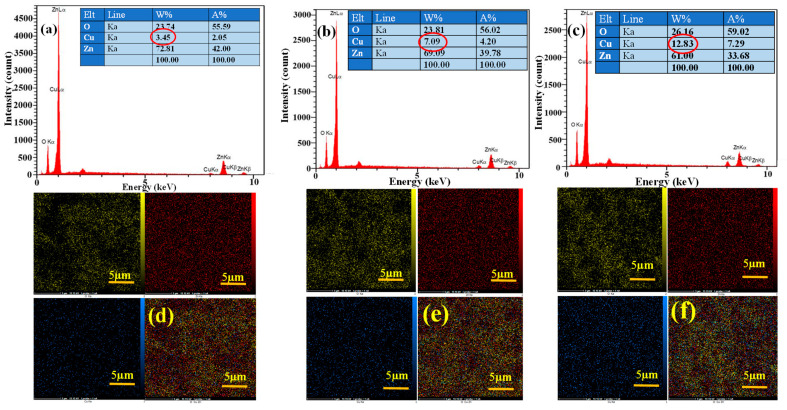
EDX spectra of ZnO-CuO nanocomposite for (**a**) ZC5, (**b**) ZC10, and (**c**) ZC20, and mapping analysis for (**d**) ZC5, (**e**) ZC10, and (**f**) ZC20.

Figure 6a–c presents the EDX elemental analysis results for ZnO NRs with initial Cu layer thicknesses of 5, 10, and 20 nm, respectively. All expected elements, namely Zn, O, and Cu, are detected in the EDX analysis. For ZnO NRs with initial Cu layer thicknesses of 5, 10, and 20 nm, the weight percent of Cu was 3.45, 7.09, and 12.83, respectively. As predicted by the quantitative analysis, increasing the thickness of the Cu layer increased the amount of Cu on the surface of ZnO NRs. Corresponding EDX mapping analysis results are presented in Figure 6d–f for ZnO NRs with initial Cu layer thicknesses of 5, 10, and 20 nm, respectively. The EDX mapping results show the uniform distribution of different elements in the synthesized material.

### 3.2. Optimal Sensing Temperature

**Figure 7:**
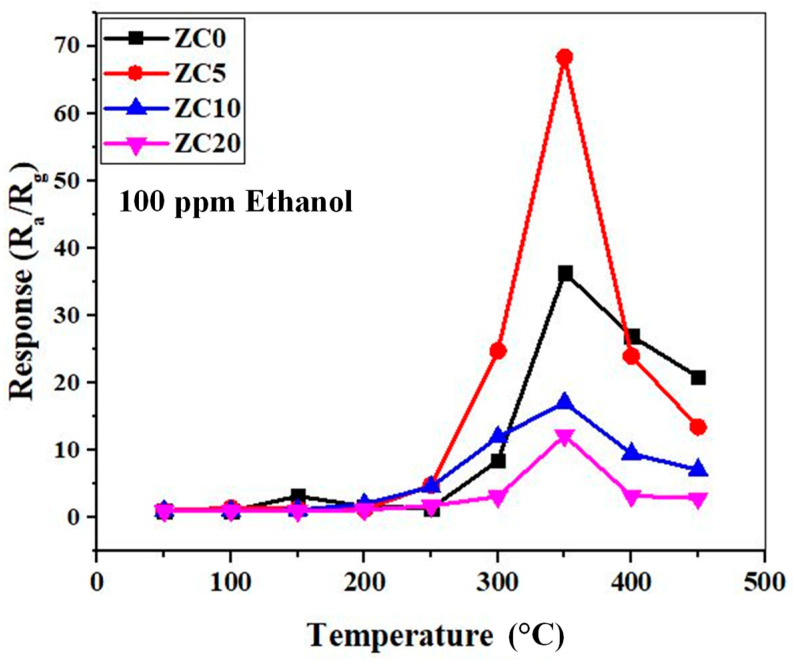
Response of pristine and CuO-decorated ZnO NR gas sensors to 100 ppm ethanol as a function of the sensing temperature.

As the first step, we tried to find the optimal sensing temperature of the fabricated gas sensors. To this end, the sensors were exposed to 100 ppm ethanol gas at various temperatures, and the obtained results are presented in Figure 7. All gas sensors show the same trend. Initially, the response is low, but it gradually increases with the increasing temperature, reaching a maximum at a certain temperature. Finally, with a further increase in the sensing temperature, the response decreases. The optimal sensing temperature for all gas sensors is 350 °C. The results show that the sensor with an initial Cu layer thickness of 5 nm (ZC5) has the highest response (resistance change) to the ethanol gas.

**Figure 8:**
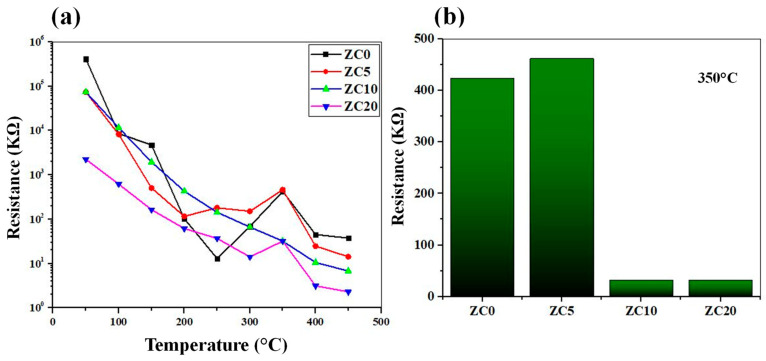
(**a**) Air resistance values of different gas sensors as a function of temperature. (**b**) Air resistance of gas sensors at 350 °C.

Figure 8a shows the variations in the sensors’ resistances as a function of temperature. In general, the resistance values decrease with an increase in temperature. This is due to the jumping of electrons from the valence band of the sensing materials to the conduction band, leading to increased conductivity. However, in some cases, at higher temperatures, the resistance increases, which is due to the adsorption of ionic oxygen species on the surface of the gas sensor, leading to an increase in the resistance. Moreover, Figure 8b shows the air resistance values of all gas sensors at optimal sensing temperatures. Upon the decoration of CuO on ZnO, the resistance increases due to the transfer of electrons from ZnO to CuO. However, with the increase of the initial CuO layer to 10 and 20 nm, the resistance decreases. This may stem from the fact that CuO has a more dominant role in the overall resistance of samples, even though the samples still show n-type conductivity.

### 3.3. Sensor Responses

**Figure 9:**
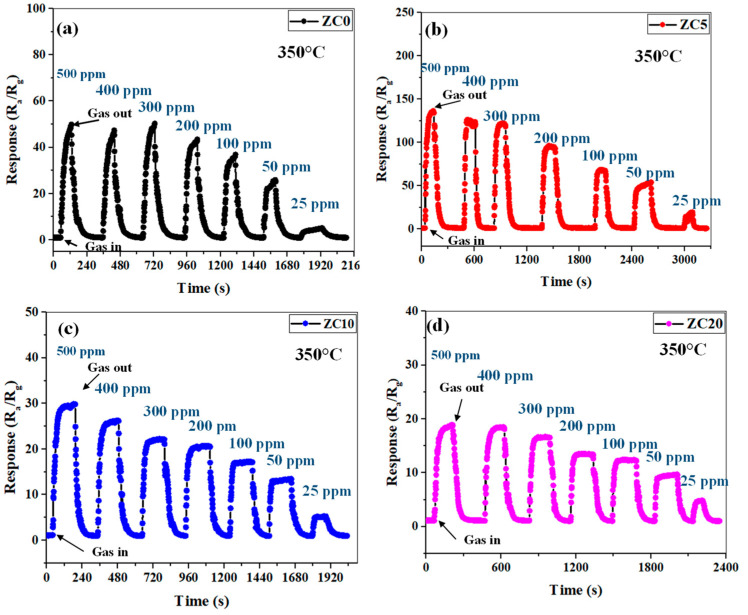
Dynamic response of (**a**) ZC0, (**b**) ZC5, (**c**) ZC10, and (**d**) ZC20 gas sensors to various concentrations of ethanol at 350 °C.

In this section, the sensor performance when exposed to different concentrations of ethanol was studied. The responses of the pristine and CuO-decorated gas sensors to 25 ppm to 500 ppm concentrations of ethanol at 350 °C are shown in Figure 9a–d. This figure revealed that all the fabricated sensors can measure different concentrations of ethanol gas, but their performances are different. The best resistance change is provided by the ZC5 sensor.

**Figure 10:**
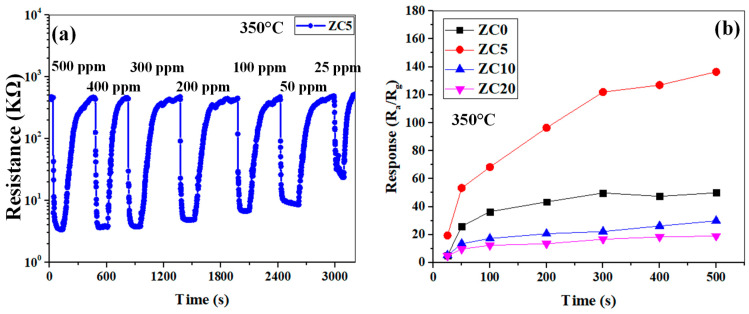
(**a**) Dynamic resistance curve of ZC5 gas sensors to various concentrations of ethanol at 350 °C. (**b**) Calibration curves of pristine and CuO-decorated ZnO gas sensors to 25–500 ppm ethanol at 350 °C.

Figure 10a shows the dynamic resistance curve of the ZC5 gas sensor for different concentrations of ethanol at 350 °C. Upon injection of ethanol gas, the resistance is decreased, demonstrating the n-type behavior of the gas sensor. The resistance variations illustrate a good recovery in all ethanol concentrations for the reported sensor, which means that the ethanol is injected into the test chamber after the sensor has returned to its rest condition. The calibration curves of all the fabricated gas sensors are presented in Figure 10b. If we define sensitivity as the slope of the calibration curve, the ZC5 gas sensor shows the highest sensitivity to ethanol gas among other gas sensors. Its responses to 25, 50, 300, and 500 ppm ethanol were 19.4, 53.4, 122, and 136.46, respectively.

**Figure 11:**
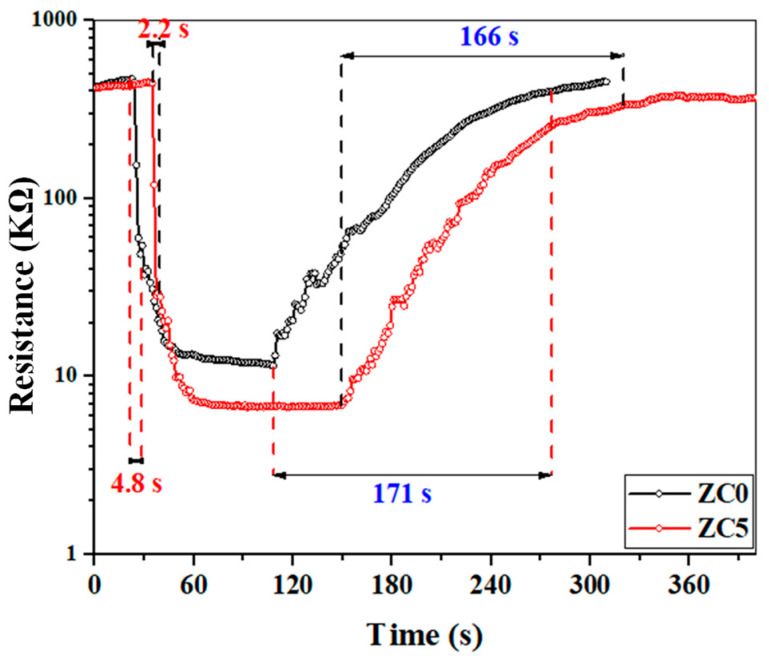
Response and recovery times of ZC0 and ZC5 gas sensors to 100 ppm ethanol at 350 °C.

Figure 11 shows the dynamic response curves of ZC0 and ZC5 gas sensors to 100 ppm ethanol at 350 °C. The response time and the recovery time for the ZC0 gas sensor are 4.8 and 171 s, respectively. These values for the ZC5 gas sensor are 2.2 and 166 s, demonstrating the faster dynamics of the ZC5 gas sensor.

**Figure 12:**
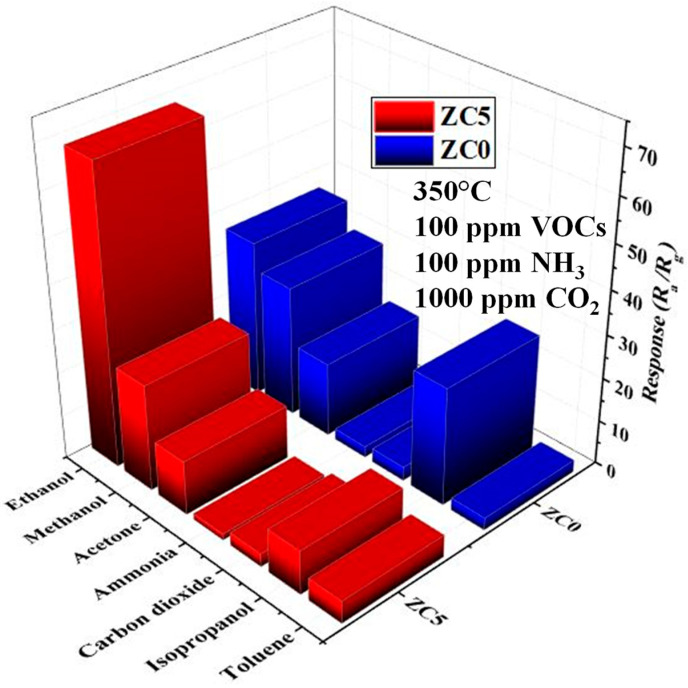
Selectivity patterns of ZC0 and ZC5 gas sensors to different gases at 350 °C.

**Figure 13:**
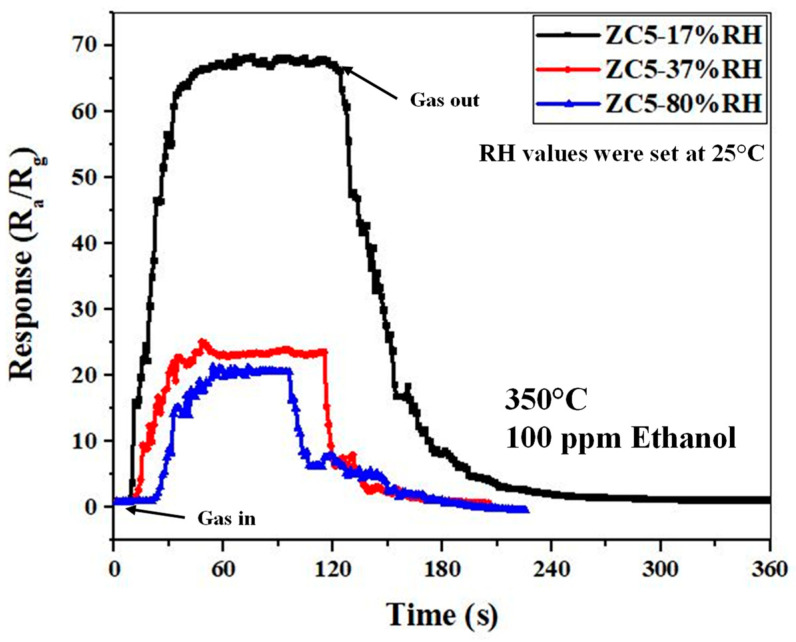
Dynamic response of optimized gas sensor to 100 ppm ethanol in the presence of various levels of humidity.

Figure 12 shows the responses of the ZC5 and the ZC0 gas sensors to 100 ppm of ethanol vapor (C_2_H_5_OH, 99.99%), methanol vapor (CH_3_OH, 99.5%), acetone vapor (C_3_H_6_O, 99.8%), isopropanol vapor (C_3_H_8_O, 99.5%), toluene vapor (C_7_H_8_, 99%), 100 ppm of ammonia (NH_3_, 96%) gas, and 1000 ppm CO_2_ (99.99%) gas at 350°C. As can be seen, the response of the ZC5 gas sensor to ethanol is higher than other gases, demonstrating its good selectivity. Furthermore, the ZC5 gas sensor showed much better selectivity compared with the ZC0 gas sensor. Figure 13 shows the dynamic response of the optimized gas sensor to 100 ppm ethanol in the presence of various levels of humidity. A sensitive RH probe was used to measure the actual temperature and RH value of the mixed humid gas before it was loaded into the chamber. The temperature of the humidified gas mixture was measured at about 25 °C. During the sensing tests, the heater temperature under the sensor samples was set at 350 °C. As can be seen, by increasing the humidity, the response is decreased. This is due to the fact that in the presence of high humidity, the water molecules will occupy the active adsorption sites on the sensor and thus limit the number of available sites for incoming ethanol gas molecules, leading to a decrease in the sensor’s response in the presence of humidity. As can be seen, even at such a relatively high operating temperature, the response in the presence of humidity is significantly decreased. Some possible strategies to decrease the negative effect of the water vapor on the gas response are increasing the hydrophobicity of the sensing layer and using a filter to prevent water molecules from reaching the sensor surface.

**Figure 14:**
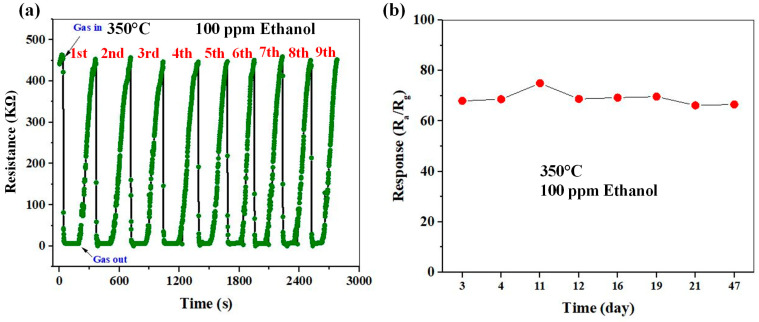
(**a**) Repeatability and (**b**) long-term stability of the ZC5 sensor to 100 ppm ethanol gas at 350 °C.

Figure 14a shows the repeatability of the ZC5 sensor, measured for nine sequential cycles of exposure of the same sensor to 100 ppm ethanol gas at 350 °C. As shown, the sensing curves are almost similar, and the response values are very close together, verifying the good repeatability of the gas sensor.

Long-term stability is one of the main parameters for gas sensors in practical applications. Generally, metal oxide-based gas sensors show good long-term stability [38,39]. In the present study, since the sensing temperature of the gas sensor is relatively high, it is necessary to study its long-term stability, as high operating temperatures can accelerate the process of degradation and aging of the sensor material. Figure 14b represents the long-term stability of the ZC5 sensor when exposed to 100 ppm ethanol at 350 °C for 47 days. There is a negligible variation in the gas response, showing the good stability of the fabricated gas sensor despite a relatively high sensing temperature.

Table 1 compares the results obtained in this study with previous works in the literature. As can be seen, the proposed optimized gas sensor in this study shows high sensitivity and fast response time in comparison with its counterparts in other works. Gas sensors based on metal oxides such as ZnO often exhibit high operating temperatures. As shown in Table 1, several sensors in the literature work at temperatures higher than 300 °C [40,41]. In this study, the optimal sensing temperature was 350 °C. For practical applications, it can lead to relatively high power consumption. Some strategies to lower the optimal sensing temperatures, such as noble metal decoration [42], the use of UV light [43], operation of the sensor in self-heating mode [44], and irradiations with high energy ions [45], can be applied to it in future studies to decrease the sensing temperature.

## 4. Gas Sensing Mechanism

In this study, the sensors showed an n-type behavior. The sensing mechanism can be explained in terms of resistance changes and the formation of the electron depletion layer (EDL). Since CuO has not fully covered the whole ZnO surface, some parts of the ZnO NRs are directly exposed to air. At the beginning of the measurement, when the sensor is exposed to air, oxygen molecules will be adsorbed on the surface of the materials, forming O_2_^−^, O^−^, and O^2−^ ions by capturing electrons from the conduction band of ZnO as follows [52,53,54,55]:

O_2_ (gas) → O_2_ (ads) (7)

O_2_ (ads) + e^−^ → O_2_^−^ (ads) (100 °C < T) (8)

O_2_^-^ (ads) + e^−^ → 2O^−^ (ads) (100 °C < T < 400 °C) (9)

O^−^ (ads) + e^−^ → O^2−^ (ads) (400 °C < T) (10)

As a result, EDL will be formed on the outer surfaces of the sensing material. In this state, the resistance of the gas sensor is higher than that in the vacuum condition, where there are no oxygen ions to be adsorbed on the surface of the gas sensor. When ethanol is injected into the gas chamber, it reacts with already-adsorbed oxygen ions on the surface of the gas sensor as follows [47,50,56,57]:

C_2_H_5_OH (gas) + 6O^−^_(ads)_ → 2CO_2_ + 3H_2_O + 6e^−^ (11)

As a result, the electrons will be liberated, and the thickness of EDL will narrow. Consequently, the resistance decreases upon exposure to ethanol gas, resulting in the appearance of a sensing signal.

**Figure 15:**
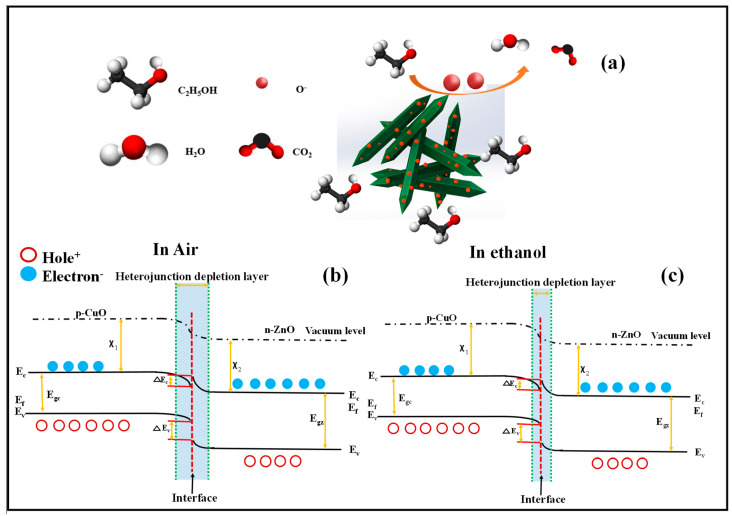
(**a**) Schematic representation of ethanol sensing mechanisms of CuO-decorated ZnO NRs. Ethanol sensing mechanism of CuO-decorated ZnO NRs in (**b**) air and (**c**) in ethanol atmosphere.

The specific question about our results is why the optimized gas sensor showed an enhanced gas response in comparison to the pristine ZnO gas sensor and why 5 nm copper deposition led to an optimized decoration with the highest sensor response. Figure 15a shows the graphical schematic of the ethanol gas sensing mechanism for CuO-decorated ZnO NRs. As shown, CuO NPs are loaded evenly on the surface of ZnO NRs, leading to the formation of heterojunctions between p-CuO and n-ZnO. The band gaps and the electron affinities of CuO and ZnO are (E_gc_ = 1.35 eV, χ_1_ = 4.07 eV) and (E_gz_ = 3.37 eV, χ_2_ = 4.35 eV), respectively. Due to the n-type nature of ZnO, in intimate contact, the electrons will flow to CuO to equate the Fermi levels on both sides of the contacts. The resulting heterojunction in the air is shown in Figure 15b. The barrier heights of the conduction band (∆E_C_ = χ_2_ − χ_1_) and valence band [E_V_ = (E_gz_ − E_gc_) − ∆E_C_] at the p–n junction are 0.28 eV and 1.74 eV, respectively. Upon exposure to ethanol gas, the electrons will return to the surface of the gas sensor and the thickness of the EDL will decrease, as shown in Figure 15c [29,56]. As shown in Figure 6, among CuO-decorated gas sensors, the sensor with an initial Cu thickness of 5 nm showed the highest gas response. Further increases in the initial Cu thickness decreased the response of the gas sensor. This can be due to the decrease of the ZnO adsorption sites on ZnO NRs upon CuO decoration. As we know, ZnO has better intrinsic gas sensing properties than CuO. For the sensors with a thickness of Cu > 5 nm, the surface of ZnO NRs is mostly covered by CuO, which causes a reduction in the exposed areas of ZnO compared with the sensor with an initial Cu thickness of 5 nm.

## 5. Conclusions

Pristine and CuO-decorated (5, 10, and 20 nm thick initial Cu layer) ZnO NRs were prepared for ethanol gas sensing studies. The characterization techniques such as XRD, SEM, and EDS demonstrated the formation of CuO nanoparticle-decorated ZnO NRs with the desired morphology and chemical composition. By establishing gas sensing measurements at different temperatures (50–450°C) for different gas sensors, the optimum temperature and the optimum initial copper layer thickness for decoration were obtained. It was found that the sensor with an initial Cu layer of 5 nm showed the highest response to this gas. Its response at 350 °C to 50 ppm ethanol was 53.4. Additionally, the response time and recovery time to 100 ppm ethanol gas were 2.2 and 166 s, respectively. Furthermore, the optimized gas sensor exhibited selectivity, good repeatability (during nine sequential cycles), and long-term stability (47 days) for ethanol gas. Improved gas response was attributed to the presence of an optimized amount of CuO on the surface of ZnO as well as the formation of p-CuO/n-ZnO heterojunctions.

## Figures and Tables

**Table 1:** Comparison between ethanol gas sensing response of optimized gas sensor in this study and those reported in the literature.

Sensing Materials	Conc. (ppm)	T (°C)	Response (R_a/_R_g_ or R_g_/Ra)	Response Time (s)	Recovery Time (s)	Ref.
ZnO-CuO nanowires	100	300	28	2	72	[29]
ZnO-CuO-decorated g-C_3_N_4_	500	260	16	87	169	[46]
ZnO-CuO flower-like	100	300	98.8	7	9	[27]
ZnO-CuO	20	320	7	6	36	[47]
Neck-connected ZnO	50	375	130 (I_g_/I_0_)	120	70	[40]
ZnO-CuO	100	300	~1.5	-	-	[48]
Al-doped ZnO/CuO	100	25	131.1	-	-	[49]
ZnO-CuO	80	122	44	22	99	[50]
Ag-functionalized CuO nanoribbons	100	25	2.6	2	-	[51]
ZnO flower-like	400	350	31	10	4	[41]
CuO-decorated ZnO NRs	100	350	68.7	2.2	166	This work

## Data Availability

Not applicable.
